# WhiteRef: A New Tower-Based Hyperspectral System for Continuous Reflectance Measurements

**DOI:** 10.3390/s150101088

**Published:** 2015-01-08

**Authors:** Karolina Sakowska, Damiano Gianelle, Alessandro Zaldei, Alasdair MacArthur, Federico Carotenuto, Franco Miglietta, Roberto Zampedri, Mauro Cavagna, Loris Vescovo

**Affiliations:** 1 Sustainable Agro-Ecosystems and Bioresources Department, Research and Innovation Centre—Fondazione Edmund Mach, Via E. Mach 1, 38010 S. Michele all'Adige (TN), Italy; E-Mails: karolina.sakowska@fmach.it (K.S.); damiano.gianelle@fmach.it (D.G.); roberto.zampedri@fmach.it (R.Z.); mauro.cavagna@fmach.it (M.C.); 2 Meteorology Department—Poznan University of Life Sciences, Piatkowska Street 94, 60-649 Poznan, Poland; 3 Foxlab Joint CNR-FEM Initiative, Via E. Mach 1, 38010 S. Michele all'Adige (TN), Italy; E-Mails: federico.carotenuto@fmach.it (F.C.); f.miglietta@ibimet.cnr.it (F.M.); 4 Institute of Biometeorology, CNR, 50145 Firenze, Italy; E-Mail: a.zaldei@ibimet.cnr.it; 5 NERC Field Spectroscopy Facility, School of Geosciences, University of Edinburgh, EH9 3JW Edinburgh, UK; E-Mail: alasdair.macarthur@ed.ac.uk; 6 Institute of Ecology—University of Innsbruck, Innsbruck A-6020, Austria

**Keywords:** hyperspectral system, proximal sensing, biophysical parameters, carbon fluxes, validation

## Abstract

Proximal sensing is fundamental to monitor the spatial and seasonal dynamics of ecosystems and can be considered as a crucial validation tool to upscale *in situ* observations to the satellite level. Linking hyperspectral remote sensing with carbon fluxes and biophysical parameters is critical to allow the exploitation of spatial and temporal extensive information for validating model simulations at different scales. In this study, we present the WhiteRef, a new hyperspectral system designed as a direct result of the needs identified during the EUROSPEC ES0903 Cost Action, and developed by Fondazione Edmund Mach and the Institute of Biometeorology, CNR, Italy. The system is based on the ASD FieldSpec Pro spectroradiometer and was designed to acquire continuous radiometric measurements at the Eddy Covariance (EC) towers and to fill a gap in the scientific community: in fact, no system for continuous spectral measurements in the Short Wave Infrared was tested before at the EC sites. The paper illustrates the functioning of the WhiteRef and describes its main advantages and disadvantages. The WhiteRef system, being based on a robust and high quality commercially available instrument, has a clear potential for unattended continuous measurements aiming at the validation of satellites' vegetation products.

## 1. Introduction

The understanding of carbon cycle dynamics in terrestrial ecosystems—in a climate change context—is of the highest scientific and socio-economic importance. Biophysical parameters and ecosystem carbon fluxes can be measured using different methods, and at different spatial and temporal scales. The Eddy Covariance (EC) technique [1–3] has become a standard method and it was adopted within the FluxNet and the other carbon observation networks, including the European Integrated Carbon Observation Network (ICOS).

Linking the ecosystem fluxes measured by means of the EC technique (characterized by a generally modest spatial coverage—the area sampled by this technique varies between tens of meters and few kilometers) and remotely sensed information is considered one of the most promising methods for scaling up the surface fluxes [4–6]. However, a significant effort is required to build a consistent network to link long-term fully validated *in situ* data with aircraft and satellite level observations.

The ground-based approach can provide a truly deeper insight into spectral proxies which enable monitoring the processes regulating carbon and water fluxes exchanged between terrestrial ecosystems and the atmosphere. *In situ* optical sampling, can provide high spatial, temporal, and spectral resolution unique datasets [6].

In the spatial domain, *in situ* observations allow scale-appropriate observations, which are able to match the spatial footprint of the EC towers. Also, in the temporal domain, such observations can be carried out on a continuous basis, hence providing high resolution data [7]. Considering the spectral resolution, the use of hyperspectral sensors allows the remote discrimination of subtle signals that are directly associated with plant biophysical characteristics and physiology. Such high-dimensional data generated by hyperspectral sensors create novel scientific opportunities for innovative analysis techniques in the frame of EC and flux studies.

**Figure 1.**
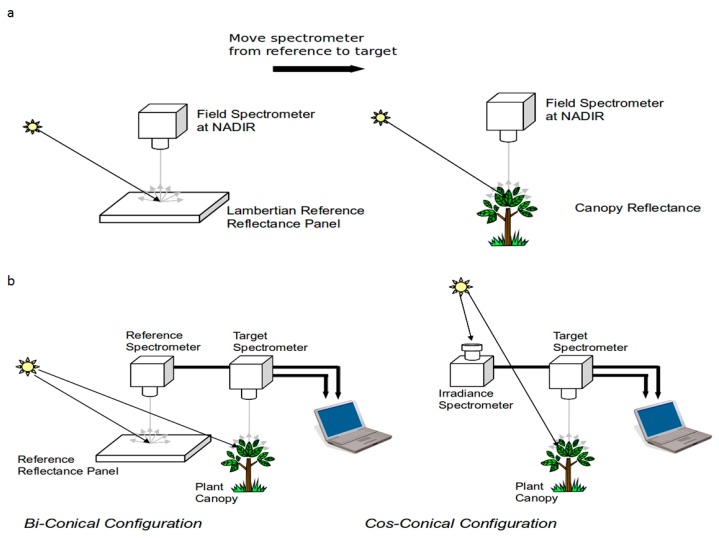
Single Field of View (SFOV, scheme (**a**)) and Dual Field of View (DFOV, scheme (**b**)) systems (with permission, copyright of NERC, Field Spectroscopy Facility, University of Edinburgh, 2009).

The already existing instrument setups for continuous *in situ* hyperspectral observations within the optical sampling community (COST Action ES0903 EUROSPEC, SpecNet network [6,7]) are quite diverse, and they are based on two main approaches (Figure 1): single field-of-view mode (SFOV), and dual field-of-view (DFOV) mode [7,8].

In the SFOV mode, first measurements are taken over a reference standard (most commonly a white reference panel), and they are immediately followed by a measurement of the target surface. Relative reflectance at each band is computed by dividing target radiance by reference radiance. This is a relatively simple approach (only one instrument with a single fiber is needed), although it is clear that to acquire a sequence of target-white reference measurements, a moving part is needed (either the fiberoptic head or the white reference panel [9]). Also, one of the major issues of this approach (and of the DFOV in the bi-conical mode) is related to the possible degradation of the white reference panel, which needs to be placed in the field throughout the whole growing season. Another issue to be addressed regarding the SFOV is that, due to the non-simultaneous measurements of the target and the white reference—in unstable weather conditions—the changing illumination can lead to significant errors in the obtained reflectance values.

DFOV mode requires either a dual fiber spectrometer or a pair of spectrometers, and for this reason it tends to be more complex and expensive, although no moving part is generally needed. The DFOV can be used both in the bi-conical and in the cos-conical mode (Figure 1). In the first case, one spectrometer head is looking at a white panel and the obtained radiance is used as a reference to calculate reflectance, while in the second case the reference head (with a cosine diffuser fitted) is pointing towards the sky in order to measure solar irradiance. The DFOV mode is a very common approach for multispectral measurements [7] as multiband sensor are of significantly lower cost than hyperspectral spectrometers. Nevertheless, several DFOV field logging hyperspectral systems have been developed, each adopting different setups and technologies [7,10,11].

Although the cos-conical DFOV is a good standard for continuous measurements in the visible (VIS) and Near Infrared (NIR) regions, there are some serious issues when measuring in the Short Wave Infrared (SWIR) domain, caused by the cosine response of the receptors. The performance of the cosine diffusers in this area of the spectrum is very poor and the signal can be highly attenuated for longer wavelengths (>1400 nm [12]).

Most of the optical sensors adopted at the EC towers are acquiring observations in the VIS and in the NIR wavelengths [10,11]. Green plants absorb solar radiation in the photosynthetically active radiation (PAR) spectral region (400–700 nm), which is used as an energy source for the photosynthesis process. On the other hand, leaf cells strongly scatter solar radiation in the NIR spectral region. Thus, VIS bands typically provide information on canopy chlorophyll absorption, while the NIR scattering is influenced mostly by canopy structure. Large use has been made of greenness spectral vegetation indices (VIs), and in particular of normalized difference mathematical combinations of NIR and VIS bands, which can be used (in a statistical approach) as proxies of vegetation biophysical characteristics [13–16].

At high spectral resolution, more subtle signals related to vegetation status, physiology, and structure can be detected. For instance, the Photochemical Reflectance Index—based on the green portion of the spectrum (530 and 570 nm)—demonstrated to be a valid proxy of Light Use Efficiency (LUE) both at leaf and at canopy level [17,18]. Also, the so-called “red-edge” region (the steeply sloped region of the vegetation reflectance curve, situated between 690 nm and 740 nm, and resulting from the transition from chlorophyll absorption to NIR leaf and canopy scattering) was shown to play a major role for estimating leaf chlorophyll content [19,20] and fraction of absorbed PAR (f_APAR_) [21,22]. For their ability to monitor f_APAR_ green and LUE, both red-edge indices and PRI are widely used in the carbon flux optical sampling community [5,23].

At longer wavelengths, in the “NIR shoulder” domain—which is sensitive to canopy structure—the Normalized Infrared Difference Index (NIDI) was recently investigated as a proxy of phytomass [24], while the Water Band Index (WBI)—calculated as a ratio of 900 and 970 nm bands—has been widely used in canopy water content studies [25].

The use of the SWIR region of the spectrum was proposed many years ago, and despite the very promising results [26], indices using this spectral region have been underutilized. Such indices are based on the sensitivity of canopy reflectance to water content in the 1500–1750 nm region (a decrease in leaf water content causes an increase in SWIR reflectance, and *vice versa*). Water content determination can be considered as a promising approach to estimate vegetation structural parameters (biomass, leaf area index—LAI) from remote sensing platforms. Furthermore, the SWIR bands can contribute to detect cellulose content and thus non-photosynthetic vegetation [27]. However, more studies integrating SWIR, NIR and VIS observations, using the full spectrum, are needed [28]. High spectral resolution datasets are crucial for *in situ* validation of the satellite sensors products [5] and allow a ground-based investigation of novel approaches for the estimation of biophysical characteristics.

Besides the aforementioned statistical approach, which consists on the computation of VIs or on the use of several bands in multiple regression models [29,30], research is moving towards a more physical approach involving radiative transfer models (RTM [31,32]), which link, on a physical law basis, the full-spectrum reflectance and canopy, leaf and soil properties.

In this work, we present the WhiteRef, a new SFOV hyperspectral system based on the ASD FieldSpec Pro spectroradiometer (VIS, NIR and SWIR: 350–2500 nm, Analytical Spectral Devices, Inc., Boulder, CO, USA) and designed for automatic and continuous acquisition of radiometric data at the EC research sites.

## 2. Methods

The ASD-WhiteRef system is an automatic system designed for continuous and unattended acquisition of canopy reflectance measurements. The WhiteRef system was developed by the Forests and Biogeochemical Cycles Research Group, Sustainable Agro-Ecosystems and Bioresources Department, Research and Innovation Centre—Fondazione Edmund Mach, San Michele all'Adige Italy, together with the Institute of Biometeorology—National Research Council, Firenze, Italy. The system was designed within the context of the COST Action ES0903 EUROSPEC, which was focused on the need to develop and exploit hyperspectral logging systems for continuous measurements at the EC research sites, for combining spectral and flux observations and for satellite validation purposes.

One of the objectives of this work was to develop a system that could be used to upgrade a standard, high quality spectroradiometer measuring not only in the VIS and NIR, but also in the SWIR wavelengths. As mentioned earlier, a SFOV configuration was chosen due to the poor cosine response in the SWIR wavelengths [12]. The core of the WhiteRef system is a commercially available ASD FieldSpec Pro single-beam spectroradiometer (model number FSP 350-2500P), which was upgraded for standalone operations.

The ASD-WhiteRef system was installed in May 2013 at the EC tower of the FLUXNET Monte Bondone site (IT-MBo, Italy). The study site is a permanent alpine grassland located at 1550 m a.s.l. on the Viote del Monte Bondone plateau (46°00′N, 11°02′E, Italian Alps). The vegetation of the area is dominated by *Festuca rubra* (L.) (covering 25% of the area), *Nardus stricta* (L.) (13%) and *Trifolium* sp. (L.) (14.5%), and is representative of a typical low productive meadow of the Alps. The site is managed as an extensive meadow with low mineral fertilization (applied in autumn) and is cut once a year, usually in mid-July. The maximum canopy height at the peak of the growing season (mid-June to early July) can reach approximately 30 cm [33].

The climate of this area is sub-continental (warm and wet summer) and is characterized by a mean annual temperature of 5.5 °C, with monthly averages ranging from −3.1 °C in February to 14.3 °C in July. The annual mean precipitation is 1244 mm, with maximum values in May (138 mm) and October (162 mm). The snow-free period lasts typically from early May to late October. The site is characterized by a regular East-West wind circulation, showing along this direction an almost flat topography with a homogeneous vegetated fetch of more than 500 m [33].

An experimental footprint analysis demonstrated that 30% (in stable atmospheric conditions) to 80% (in unstable conditions) of the total CO_2_ flux originates within 30 m from the EC tower. The EC system consists of a Licor Li-7500 open-path infrared gas analyzer (Li-COR Inc., Lincoln, NE, USA) and a Gill R3 3-D ultrasonic anemometer (Gill Instruments Ltd., Lymington, UK), both mounted at a height of 2.5 m. Along with EC flux measurements, the main meteorological and soil physical variables are measured, including incident total and diffuse PAR (BF3H Sunshine Sensor, Delta T Devices Ltd, Cambridge, UK), which enables the computation of the Diffusion Index (DI—the ratio between diffuse and total incident PAR) [34].

Additionally, in the growing season of 2013 the f_APAR_ and the total chlorophyll content were determined during different vegetation growing stages (eight measurement dates in the period between May and August 2013). F_APAR_ data were collected by means of the SunScan probe (Delta-T Devices Ltd., Cambridge, UK) and carried out in the footprint of the ASD WhiteRef system (in six permanently fixed measurement points, and in two repetitions). Chlorophylls were measured and characterized using the UV-VIS spectroscopy method. Green leaf samples (>30 g) were collected within the footprint of the EC tower, and nearby the footprint of the ASD WhiteRef system. Collected samples were immediately stored in sealed plastic bags and were kept fresh in an ice chest until transported to the laboratory. In the first step the green tissue samples were grinded in the presence of liquid nitrogen. Next, 15 randomly chosen subsamples were immersed in 80% acetone (0.1 g per 10 mL), shaken for 10 min in an automatic shaker at 250 rpm (Universal Table Shaker 709; ASAL S.r.l., Cernusco sul Naviglio, Milano, Italy), and centrifuged at 4000 rpm for 10 min (Eppendorf 5810 R; Eppendorf Italia S.r.l., Milano, Italy) in order to remove particles from the solution. The absorbance of the extracted solutions was measured at 470, 646.8 and 663.2 nm by the UV/VIS Shimadzu UV-1601 spectrophotometer (Shimadzu Italia S.r.l., Milano, Itlay), and the concentrations of chlorophyll a and chlorophyll b were calculated with equations given for 80% acetone solvent (where the pigment concentrations are given in μg·ml^−1^) [35]. The weight of sampled sediment was used to calculate pigments concentrations per unit leaf mass (mg·g^−1^) and the weight of green biomass per m^−2^ (obtained by harvesting and separating three randomly chosen plots of 1 m^2^ located within EC footprint) was used to compute the total chlorophyll (a + b) content in (mg·m^−2^).

### 2.1. Hardware

**Figure 2.**
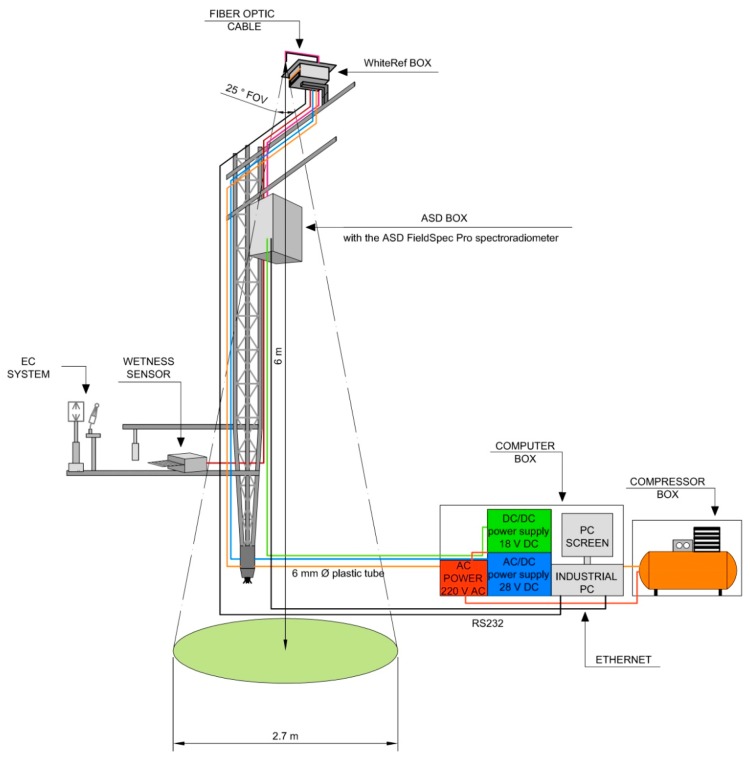
Schematic drawing of the WhiteRef hyperspectral system deployed at the EC tower.

Figure 2 is illustrating the ASD-WhiteRef system parts. The system hardware consists of three main components:

(I) the WhiteRef box
(II) the ASD box
(III) the computer box

The power consumption of the whole WhiteRef system is of 200 W in operation, and 20 W in a standby mode.

#### 2.1.1. The WhiteRef Box

**Figure 3.**
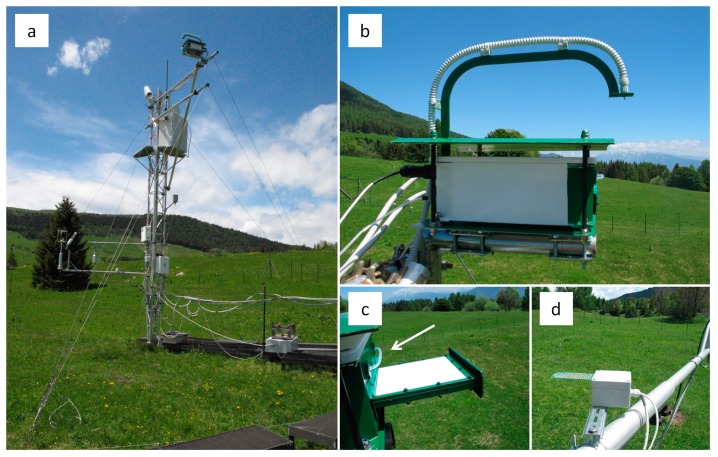
Viote del Monte Bondone research site: (**a**) Eddy Covariance tower equipped with the ASD WhiteRef hyperspectral system; (**b**) WhiteRef box; (**c**) white reference panel during the measurements (the plastic pipe blowing compressed air is indicated by the white arrow), (**d**) wetness sensor.

The WhiteRef box (weight = 3 kg, Figures 2 and 3b) is provided with a solar shield—preventing the overheating of its electronic components—and is mounted on a horizontal arm installed on the EC tower at a height of 6 m (Figure 2). This waterproof box containing the white reference panel (WR, 12.7 × 12.7 cm, ODM98-MP06, Gigahertz-Optik GmbH, Türkenfeld, Germany) was designed to enable alternative observation of the reference and the vegetation target. The WR panel and vegetation canopy radiance readings are acquired from nadir using a 5 m long bare fiber optic with a field of view of 25°. As the canopy plane is observed from a distance of 6 m, the optical canopy footprint diameter is of about 2.7 m (Figure 2).

The acquisition scheme is WR1-VE-WR2 (measurement of the radiance of the white reference panel—measurement of the canopy radiance spectra—measurement of the radiance of the white reference panel, respectively). The WR panel stored in the WhiteRef box is mounted on a mobile platform operated by means of a step motor. The WhiteRef aims at keeping the WR clean and protecting it from light, dust, rain, insects and adverse weather conditions. In fact, the WR is ejected from the box only during the measurements (Figure 3c), and each acquisition is preceded by a reading of a dedicated wetness sensor signal (Figures 2 and 3d). In case of rainfall or dew, the measurements are not performed and the WR panel remains inside the WhiteRef box. In addition, during each ejection and insertion phase, in order to remove eventual dust/insects from the measurement surface, the WR is sprayed with compressed air (5 bar) provided by a standard 100 L compressor hosted in a box at the base of the tower (Figure 2). A homogeneous air spray was obtained by installing a 6 mm plastic pipe above the WR panel, provided with a series of micro holes (Figures 2 and 3c). The ASD WhiteRed box is powered with a 28 V DC power source unit stored in a waterproof box located at the ground level. The WhiteRef box interface board allows the communication with a dedicated industrial computer (MPL PIP7 PC, MPL AG, Dättwil, Switzerland) through a serial RS232 port (Figure 2).

In order to test the efficiency of the WhiteRef box in protecting the WR panel from degradation, laboratory measurements of the adopted panel were carried out by the Field Spectroscopy Facility (FSF, Edinburgh, UK). The panel's reflectance factors were measured in an optical instrument calibration laboratory darkroom before and after field use. An ASD FS3 field spectrometer, comparable with the one used to perform the field measurements, was used to make the measurements in the darkroom. A 1000 W Tungsten Halogen lamp with a Perkin Elmer stabilized power supply was used as a light source. At the same time as the WR panel was measured, a calibrated standard Spectralon reference panel was measured under the same measurement configuration and illumination conditions and all WR panel measurements were subsequently converted to reflectance factors. The same calibrated reference panel was used and the calibration of this panel was maintained by FSF and referenced to a common source.

#### 2.1.2. The ASD Box

The waterproof and ventilated (2 × Sunon 12 V DC fans) ASD box (Figures 2 and 3a) was mounted on the upper part of the EC tower, housing the ASD FieldSpec Pro spectroradiometer (wavelength range between 350 and 2500 nm; spectral resolution—FWHM of a single emission line—of approximately 3 nm around 700 nm; sampling interval 1.4 nm: 350–1000 nm, 2 nm: 1000–2500 nm; resulting spectrum interpolated to 1 nm intervals). The operating temperature range of the ASD instrument is between 0 °C and 40 °C, while the observed air temperature range in the study site varied between 6 °C and 25 °C during the operation period of the instrument.

The spectroradiometer consists of an array of 3 spectroradiometers. The first (VNIR) refers to the 350–1050 nm range, while SWIR1 and SWIR2 ranges are 900–1850 and 1700–2500 nm, respectively. The splice between the VNIR spectrometer and the SWIR1 spectrometer is, for the adopted spectrometer, at 964/965 nm, where the response of the SWIR1 spectrometer is superior to that of the VNIR photodiode array. The splice between SWIR1 and SWIR2 turns out to occur at 1769/1770 nm.

The instrument is powered by an 18 V DC power source unit stored in a waterproof box located at the ground level. The communication between the spectroradiometer and the industrial PC is ensured by the ASD Smart Ethernet Adapter [36] enabling Ethernet connectivity using the TCP protocol (Figure 2).

#### 2.1.3. The Computer Box

The computer box is mounted at the bottom of the tower and contains: (I) the Windows XP SP2 industrial PC (MPL PIP7 PC, MPL AG, Dättwil, Switzerland) operating the WhiteRef system using a dedicated LabVIEW software and storing acquired radiometric data; and (II) the AC/DC and DC/DC power converters of 28 V (WhiteRef box) and 18 V (ASD FieldSpec Pro), respectively (Figure 2).

### 2.2. Software and Operation

**Figure 4.**
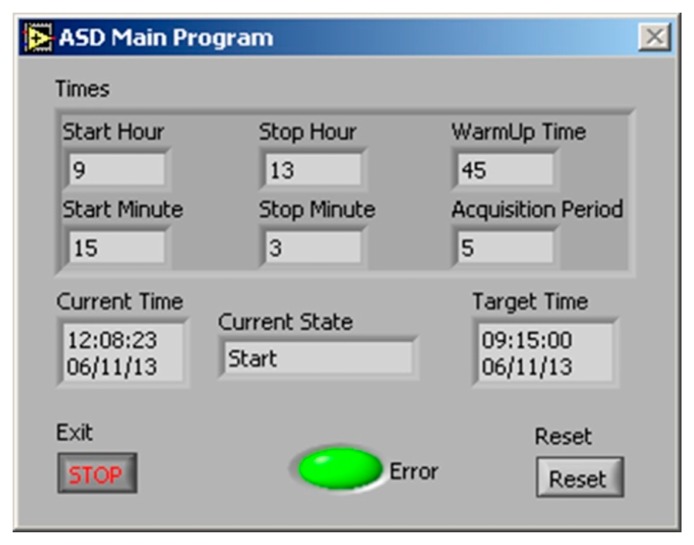
The user-friendly interface of the LabVIEW acquisition software. The acquisition start hour, stop hour and warm up time can be easily setup and the status is displayed.

The WhiteRef is operated automatically through a dedicated LabVIEW software. As the degradation rate of the WR panel was unknown at the time of installation, the system was used, on a preliminary basis, only for 3 h per day in order to limit the WR exposure to sun and to limit the effect of air pollution, thus to ensure a sufficient data quality. Data were acquired every 5 min between 10:00 a.m. and 1:00 p.m. local solar time and stored on the industrial PC. Beyond these 3 h, the system was off, although the acquisition time window can be easily changed (Figure 4).

Every start of the measurements was preceded by a warming up period of the ASD FieldSpec Pro spectroradiometer (45 min, chosen according to the manuals), which means that the instrument was powered on at 9:15 a.m. of local solar time. The spectroradiometer was optimized before each acquisition and the acquisition scheme was WR1-VE-WR2. For each acquisition, 25 scans were acquired and their average was recorded: VNIR dark current, incident solar radiance (measured with the WR panel with known reflectance), target radiance and again incident solar radiance. The radiance of the WR panel at the time of the target measurements was estimated by linear interpolation.

The control and acquisition software was constantly running on the PC desktop and communicating with the interface board of the WhiteRef box (RS232) and with the ASD FieldSpec Pro spectroradiometer (Ethernet) sending/receiving commands from both devices, which controlled the operation of the radiometer, the status of the wetness sensor, the step motor, the position of the WR panel during the measurements procedure, and the ON/OFF valve of the air compressor.

In order to provide a simple example of how hyperspectral datasets can be efficiently used for monitoring seasonal trends of vegetation biophysical parameter characteristics—such as e.g., total chlorophyll content and f_APAR_—the Red Edge Chlorophyll Index—CI_red-edge_ [5] and the Normalized Difference Water Index—NDWI [26] were calculated as follows:

(1) $$\begin{array}{c} {\text{CI}}_{\text{red}-\text{edge}}=\left(\text{R}783\right)/\left(\text{R}705\right)-1\\ \text{NDWI}=\left(\text{R}865-\text{R}1610\right)/\left(\text{R}865+\text{R}1610\right) \end{array}$$

where R705, R783, R865, R1610 are the reflectance values at 705, 783, 865 and 1610 nm, which correspond to the SENTINEL-2 central wavelengths.

## 3. Results and Discussion

As mentioned in the Methods section, laboratory measurements of the adopted WR panel were carried out before and after the measurement season. Considering the high number of measurements performed in the field by the WhiteRef system (36 acquisitions per day, each one with two panel ejections), and the fact that it was used for 5 months, a slight change in reflectance was expected.

**Figure 5.**
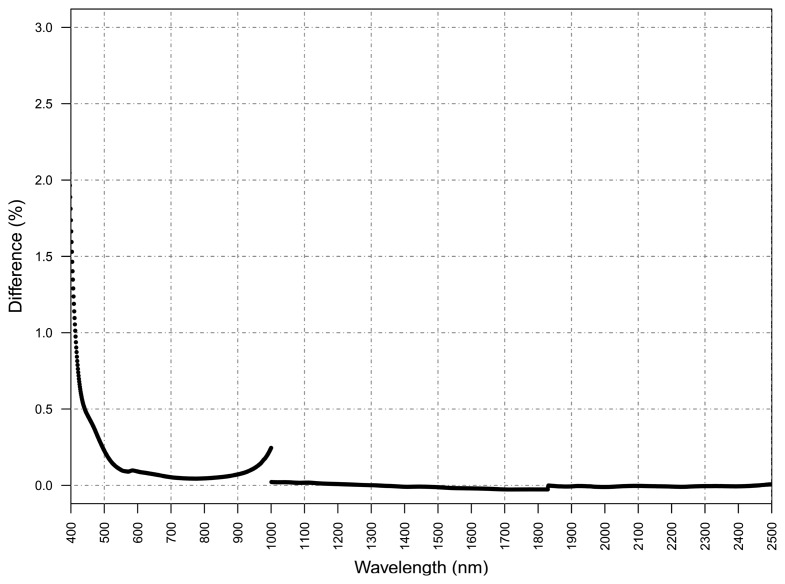
Percentage difference in reflectance of the adopted white reference panel before and after the measurement season.

Figure 5 is showing the reflectance difference (in percentage) before and after the panel was used in the field. Surprisingly, the reflectance did not show any relevant change in most of the spectrum.

A degradation of less than 2% at 400 nm was observed, signification less than that at all other wavelengths and with a detector join anomaly common with ASD full wavelength spectrometers at around 1000 nm and 1850 nm. As the reference panel degradation was negligible, in the future it will be possible to extend the acquisition window in order to explore diurnal spectral dynamics.

The WhiteRef .csv output file (a sample is displayed in Table 1) included all the relevant information about the measurement date, acquisition number, measurement type, acquisition time, wavelengths and acquired radiance/reflectance values (WR1 and WR2: first and second white reference panel radiance measurement; DC: Dark Current; VE: Vegetation radiance; REF: calculated reflectance). Each acquisition was flagged with an error code (Table 1). The error code “N” stands for acquisitions performed in stable light conditions, the code “L” indicates a change of the illumination conditions (more than 10% change between the WR1 and WR2 radiance measurements), and the code “R” indicates a rain event (no measurements carried out). Error codes proved to be extremely useful for post-processing data filtering purposes. During this phase, measurements acquired during variable light conditions (“L”) can be analyzed and undergo a further quality check.

**Figure 6.**
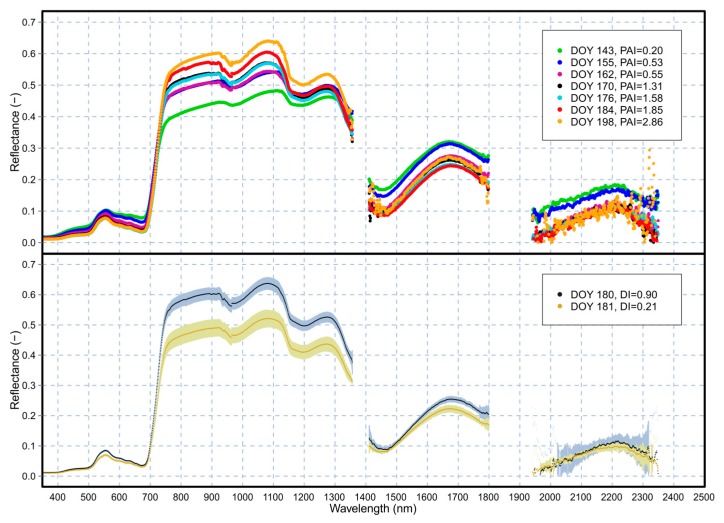
**Upper**: example of reflectance (daily average) trends starting from DOY 143 until DOY 198, with different Plant Area Index (PAI) values ranging from 0.20 to 2.86 (m^2^·m^−2^). **Lower**: reflectance under sunny (DOY 181; Diffusion Index—DI = 0.21 (−)) and cloudy conditions (DOY 180; DI = 0.90 (−)). Error bars in the lower panel represent standard deviation.

Figure 6 presents some of the daily averaged reflectance values (calculated from data collected between 10:00 a.m. and 1:00 p.m) acquired at the Viote del Monte Bondone site (the SWIR atmospheric water absorption regions around 1350 and 1900 nm and bands above 2350 nm affected by noise due to low solar irradiance, were removed [37]). Canopy reflectance (upper part of the graph) was following a typical seasonal trend, with decreasing reflectance in the VIS, and (mostly) increasing reflectance in the NIR region. On the other hand, in the SWIR domain—at 1650 nm and 2200 nm—the reflectance values were decreasing with time, due to the increasing total canopy water content and higher absorption rates [28]. The reflectance acquired on DOY 198 (lower panel of Figure 6) appears noisier in the SWIR2 region than reflectance acquired in other DOYs. This is probably due to the fact that during that day much lower and variable light conditions were recorded: 43% of acquired reflectance data were flagged as “L” (changing light conditions by more than 10% between WR1 and WR2 measurements). This accounted for more noise and the high noise level starting at a lower wavelength. The “sawtooth behavior” was not so evident with clear and stable sky conditions.

In the lower part of the graph, the effect of different light quality conditions on canopy reflectance is shown (DOY 180, DI = 0.90 (−); DOY 181, DI = 0.21 (−)). Assuming that the grassland growth within 24 h was negligible (and hence the canopy optical properties remained substantially the same), it is possible to see how DI (DI = diffuse PAR/total PAR) influenced the vegetation reflectance curve shape. For the Viote del Monte Bondone grassland, canopy reflectance was significantly increasing in the NIR when the DI values were higher, since cloudy conditions increased the canopy NIR scattering. Minor—but very important from the remote sensing point of view—changes were occurring in the green wavelengths and in the SWIR wavelengths, which were likely due to subtle changes in the xanthophyll cycle and water content status, respectively [25,38], and which will be investigated in a future study.

**Figure 7.**
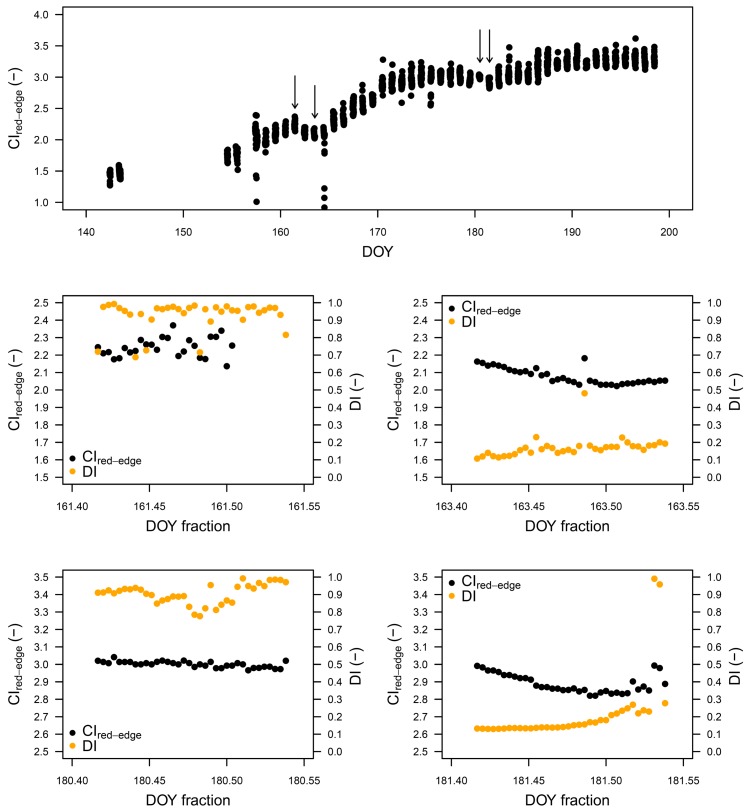
Example of Red Edge Chlorophyll Index (CI_red-edge_) seasonal and diurnal time series.

The top panel of Figure 7 shows the seasonal trend (from the beginning of the growing season to the biomass peak) of the CI_red-edge,_ an index proven to be sensitive to the amount of biomass, to f_APAR_ and canopy chlorophyll content. In the lower panels, the diurnal trends of CI_red-edge_ acquired during cloudy (left) and sunny (right) days are presented. In sunny days, it is possible to observe the typical diurnal variation of the index with a characteristic shape due to the anisotropy of canopy reflectance in the red-edge and NIR wavelengths generated by the changing position of the Sun during the measurements window (10:00 a.m. to 1:00 p.m. of local solar time). In contrary, with homogeneous cloud cover the diurnal pattern of CI_red-edge_ was not so evident due to more uniform illumination.

**Figure 8.**
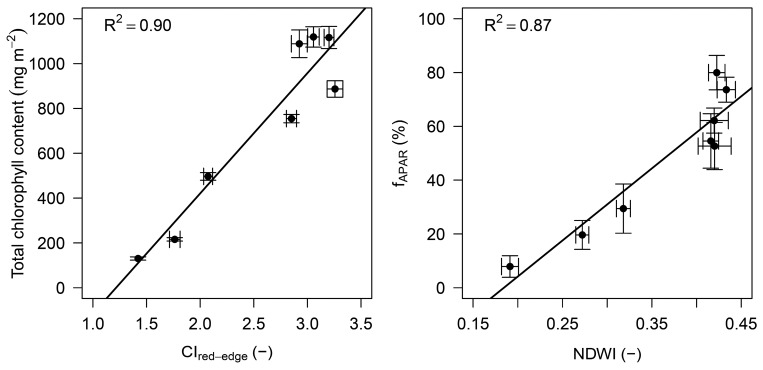
**Left**: Relationship between Red-Edge Chlorophyll Index—CI_red-edge_ (−) and total chlorophyll content (mg m^−2^); **Right**: Relationship between Normalized Difference Water Index—NDWI (−) and fraction of absorbed photosynthetically active radiation (f_APAR_). Each CI_red-edge_ and NDWI data point corresponds to a daily average calculated from reflectance data acquired between 10:00 a.m. and 1:00 p.m. *R*^2^—coefficient of determination (−), error bars represent standard deviation.

As mentioned earlier, spectral information can be used as a proxy for vegetation biophysical characteristics estimation. In Figure 8 (left panel), a simple example of how VIs are sensitive to total chlorophyll content is provided; CI_red-edge_ showed very good linear correlation with this parameter (*R*^2^ = 0.90). As demonstrated by many authors [5,15,30] the estimation of total chlorophyll content, in dynamic canopies characterized by a strong seasonal trend of LAI and f_APAR_, is a well-known approach for further estimation of Gross Primary Production.

Regarding the good potential of VIs—based on VNIR and SWIR spectral bands for capturing structural vegetation biophysical characteristic trends, Figure 8 (right panel), shows how a SWIR-based index such as NDWI is linearly correlated with the f_APAR_. The aforementioned potential of water indices is due to the fact that the SWIR absorption is strongly related to the total water content of the canopy foliage. Consequently, as grassland canopy water content (the water weight per area) relates to the quantity of above-ground biomass, thus also indirectly to LAI and f_APAR_—the water indices can be used as a proxy to retrieve various structural parameters [28].

## 4. Conclusions/Outlook

The WhiteRef system is an upgrade of a well-known, high quality, commercially available ASD spectroradiometer and it is easy to install and to manage. The WhiteRef system showed a good reliability, as no significant failures during the measurement campaigns in 2013 and 2014 were recorded. Quite unexpectedly, in the laboratory tests, the degradation of the white panel showed to be not significant, thus the protection of the WhiteRef box proved to be very efficient. However, the degradation rate will likely depend on the type of ecosystem in which measurements are conducted (dust, smog being unfavorable conditions). More studies are needed to test the system in other more challenging sites.

The LabVIEW software proved to be efficient and user-friendly. Post-processing is facilitated by the use of error flags included in the .csv output files, while a continuous data quality check is possible through an open source R software.

Thanks to the combined use of the BF3H light sensor, cloudy data can be detected and can be easily filtered, or further analyzed (e.g., for ecosystem LUE studies).

The main advantage of the presented system is that it allows the acquisition of reflectance in the SWIR wavelengths, which is an under-investigated area of the spectrum. Due to the potential of this spectral region for estimating canopy water content and structural parameters, more studies are needed within the optical sampling and the Radiative Transfer modeling communities [28].

One of the major limitations of the WhiteRef system is related to the limited FOV (25 degrees), and consequently to the limited optical footprint. This limitation has also a strong impact on the ability to provide measurements that are comparable with the eddy flux station footprint. For this reason, the system is recommendable for ecosystems with low to moderate spatial variability (e.g., grassland, crops), where the optical footprint is well representative of the satellite pixel or of the EC footprint [11]. Before the system installation, a detailed characterization and analysis of the ecosystem and the EC footprint is recommendable to check the representativeness of the spectral footprint. For this purpose, the use of Unmanned Aerial Vehicles and/or airborne remote sensing observations, adopting a spatial ecology approach, can help to highlight small-scale spatial patterns of plant traits and ecosystem functional properties [39] and to identify the best location for positioning the WhiteRef system. For observations on very heterogeneous canopies, tram systems [40], AMSPEC multiangular observation system [10] or systems such as the HyperSpectral Irradiometer (HSI) providing hemispherical reflectance [11] represent a good alternative, although such systems are not acquiring information in the SWIR region.

The WhiteRef system, which was conceived and developed mostly in Italy, aims at filling a gap in the scientific community: in fact, no system for continuous hyperspectral instruments at the EC sites including the SWIR region was tested before. For this reason, the WhiteRef system has a clear potential to become a standard tool for proximal sensing observations and *in situ* validation of reflectance and of various satellite products.

## Figures and Tables

**Table 1.** A sample of WhiteRef hyperspectral system data stored in the LabVIEW output file, including some of the acquired radiance (digital number) and reflectance (−) values for a few selected bands.

**Date**	**Acquisition**	**Measurement Type**	**Time**	**Error Code**	**R560**	**R705**	**R865**	**R1610**	**R2190**
2013-06-10	22	WR1	11:45:10	L	18,500.16	13809.13	8154.02	3705.127	1636.191
2013-06-10	22	DC	11:45:20	L	1730.117	1725.925	1723.55	0	0
2013-06-10	22	VE	11:45:29	L	3141.385	3459.879	4970.289	786.277	331.445
2013-06-10	22	WR2	11:45:42	L	16,238.52	12160.77	7462.564	3014.568	1260.846
2013-06-10	22	REF	11:45:54	L	0.09	0.154	0.534	0.234	0.229
2013-06-10	23	WR1	11:50:09	N	12,913.12	9748.673	6357.771	1895.473	720.071
2013-06-10	23	DC	11:50:20	N	1731.456	1726.593	1723.689	0	0
2013-06-10	23	VE	11:50:29	N	2785.871	3016.722	4301.75	455.641	73.753
2013-06-10	23	WR2	11:50:41	N	12,949.72	9777.406	6370.499	1891.928	790.819
2013-06-10	23	REF	11:50:53	N	0.094	0.161	0.556	0.241	0.098
2013-06-10	27	WR1	12:10:00	R	0	0	0	0	0
2013-06-10	27	DC	12:10:00	R	0	0	0	0	0
2013-06-10	27	VE	12:10:00	R	0	0	0	0	0
2013-06-10	27	WR2	12:10:00	R	0	0	0	0	0
2013-06-10	27	REF	12:10:00	R	0	0	0	0	0
